# Trauma and Transgression in Nature-Based Leisure

**DOI:** 10.3389/fspor.2021.735024

**Published:** 2021-12-14

**Authors:** Alia M. Dietsch, Everly Jazi, Myron F. Floyd, Danielle Ross-Winslow, Natalie R. Sexton

**Affiliations:** ^1^School of Environment and Natural Resources, The Ohio State University, Columbus, OH, United States; ^2^College of Natural Resources, North Carolina State University, Raleigh, NC, United States; ^3^Human Dimensions Branch, United States Fish and Wildlife Service (USFWS), Fort Collins, CO, United States

**Keywords:** trauma, transgression, nature–based activities, leisure, public lands management, outdoor recreation, constraints, barriers

## Abstract

The following article contains language, including discussion of racialized trauma, violent oppression, and more, that could trigger strong emotions and other physiological reactions. Our intent is not to retraumatize anyone, but to instead center the voices and experiences of people who have transgressed significant historical trauma and long sought lasting change and equitable outcomes for all.

## Introduction

Numerous social scientists and practitioners have sought to unearth and address multiple constraints and barriers to participation by minoritized populations in nature-based leisure activities, such as hiking, camping, hunting, fishing, and birding (Floyd, 1999, 2001; Mitchell et al., 2006; Johnson et al., 2007; Mott, 2016). The need to address constraints for these populations, particularly in the United States (U.S.), is often framed around a desire for enhanced relevancy due to the growth of population subgroups and the economic benefits generated from participation in leisure pursuits (e.g., Outdoor Foundation, 2020; State Outdoor Business Alliance Network, 2021). This frame, however, centers the dominant culture as desiring change and able to profit from increased engagement of diverse groups of people without necessarily understanding or addressing constraints to leisure that are rooted in historical trauma (Floyd, 2001; Byrne, 2012; Finney, 2014; Flores et al., 2018). Similarly, this approach often overlooks the ways in which minoritized peoples have “transgressed” oppression and created spaces for nature-based leisure despite significant historical and contemporary obstacles (hooks, 2009; Finney, 2014; Theriault and Mowatt, 2020). Discounting these traumas and transgressions erases important cultural legacies, perpetuates biases, and stymies changes needed for equitable leisure opportunities for all.

Specifically, practices and policies that institutionalized systemic racism in the U.S., including slavery, forced migration and cultural genocide, and *de jure* segregation, have legacy effects that continue to shape human interactions in public spaces (Kobayashi and Peake, 2000), including nature-based leisure contexts. For example, Taylor (2000) describes how white park and wilderness managers often display white Euro-American photographs, ideologies, and history in ways that portend personal and institutional biases which disregard the lived experiences of diverse peoples (e.g., “discoveries” of “new” places by colonizers while ignoring the names and contributions of Indigenous peoples). Thus, “preferences” for specific types of experiences and leisure pursuits are imposed by the dominant culture, controlling (inadvertently or intentionally) who and what activities are perceived as acceptable in nature-based leisure spaces (Taylor, 2018). Simultaneously, minoritized people have actively created opportunities for their own nature-based leisure outside the dominant culture's formal and informal rules (hooks, 2009; Finney, 2014; Theriault and Mowatt, 2020). These transgressions in the face of extreme duress and exclusion provide significant insight about the role of nature-based leisure in people's lives, including ways to overcome constraints on leisure participation.

Drawing upon the literature, we first describe how historical trauma can affect participation in nature-based leisure activities in the U.S. Though many types of people have been minoritized in the U.S. in relation to race, ability, sex/gender, age, and more, we specifically focus here on peoples minoritized by race or ethnicity, and nationality in the case of Native Americans (also referred to as American Indians, Indigenous peoples, and people of their respective tribal nation) who hold dual-citizenship in the U.S. and within their sovereign tribal nations. We then connect how intergenerational effects of trauma, and the power of transgressions, can shape the degree to which people seek out federal public lands where nature-based leisure activities are promoted when compatible with conservation objectives (e.g., provision of habitat for fish, wildlife, and plants). We conclude with how addressing traumas and uplifting stories of transgressions can lead to increased participation of minoritized peoples in nature-based leisure activities that typically result in improved mental and physical health outcomes. The 1948 Universal Declaration of Human Rights (UDHR) underscores the importance of leisure time to a quality life (Zandy, 2019), and research shows how leisure in nature (e.g., forests, along rivers) can significantly improve psychological and physical wellbeing (e.g., Hartig et al., 2014) in ways consistent with aspects of the United Nations New Urban Agenda (2016). Thus, expanding understanding of why minoritized populations participate in nature-based leisure and honoring stories of how these peoples have engaged with nature over time despite trauma can lead to better solutions that treat nature-based leisure as a right for all.

### Historical Trauma and Transgressions in the U.S.

Brave Heart (1998, 2003) first describes historical trauma in relation to Native Americans as the cumulative emotional and psychological wounding across generations, including the lifespan, which emanates from massive group trauma. Historical trauma for Native Americans occurred as practices of outright genocide through warfare and disease spread, forced migration from homelands, enrollment in boarding schools that punished people speaking Native languages and practicing Native customs, and state-sanctioned adoptions of Native children to non-Native families that furthered an attempt at cultural erasure (Brave Heart, 2011; Robertson, 2015). Historical trauma, as defined by Kirmayer et al. (2014), is the impact of violence, cultural suppression, and oppression of people who have a collective identity—such as a racial or ethnic background, or gender or sexual identity—beyond the generation who lived through the specific events (Kira, 2010). Thus, historical trauma affects a multitude of minoritized groups, including Black Americans in the U.S. through institutionalized oppression, such as slavery and *de jour* segregated society known as “Jim Crow”, though oppression can and does occur contemporaneously (Wiltse, 2010; Kahrl, 2012; Finney, 2014; Goodrid, 2018; Walker, 2019). Specific examples of oppression connected to historical trauma and the African American experience associated with nature-based spaces include white people tracking Black people who sought freedom from enslavement; lynching (often by hanging from trees) and beatings to punish and deter Black people for seeking freedom and equality; segregating urban park systems throughout the Jim Crow era; creating or legislating unequal facilities and access to public lands; and terrorizing or killing Black Americans in modern outdoor spaces through sanctioned forces (e.g., police) and unsanctioned vigilantes (Byrne and Wolch, 2009; Lee and Scott, 2016; Theriault and Mowatt, 2020).

Minoritized groups may be routinely (re)traumatized by historical and current events (e.g., Kobayashi and Peake, 2000) and, in response, entwine nature-based experiences with danger or cultural loss. For example, Goodrid (2018) found that historical trauma was mentioned by most Black participants (66%) interviewed about use of public lands in Texas, and that these individuals had to personally confront such fears and memories every time they engaged in nature-based leisure. As another illustration, Lee and Scott (2016) spoke with Black Americans about the normalization of a disconnect from nature due to violence targeted at Black people, to which a participant noted how trauma had “settled” in the Black community–“we just don't [participate in nature-based leisure]… that's just not what we do.” Similarly, reproducing cultural stigmas about what groups of people do (or don't do) in leisure contexts disregards historical trauma (Byrne, 2012). For example, the perception that “Black people don't swim” expunges countless incidents (including riots) across more than six decades when whites terrorized, brutalized, and outright killed Black Americans if they did swim, and used legal and, at times, violent means to dispossess Black Americans of the pools, beaches, and other swimming spots they owned or frequented and enjoyed (Wiltse, 2010; Kahrl, 2012). As a different example, recent efforts by universities and other institutions to acknowledge land dispossessions and honor Native peoples can unintentionally reinforce trauma when not accompanied by actions that right the wrongs that have occurred (Sabo et al., 2021).^1^ Contemporary land dispossessions through legal means, such as eminent domain, along the Mexico border for purposes of “national security” are similar examples of how groups oft-minoritized in the U.S. can be continually retraumatized in relation to land rights and use (Burnett and Peñaloza, 2020).

Contemporary discrimination and the threat of physical violence today reinforces the effects of historical trauma by shaping how minoritized people think about participation in nature-based leisure activities (Byrne, 2012). For example, Dr. J. Drew (Lanham, 2013), a Black wildlife ecologist, wrote of 9 rules for Black birdwatchers regarding what to expect when participating in the leisure pursuit, including “carry your binoculars—and three forms of identification—at all times, don't bird in a hoodie, nocturnal birding is a no-no.” The potential for physical harm due to overt discrimination in this context was headline news in May 2020 when a white woman called the police on Christian Cooper, a Black man who was birding in Central Park (New York), as a way of threatening him despite that she was breaking the rules by having her dog off-leash in a natural area. This incident occurred within the same month as a white police officer murdering George Floyd, a Black man (Minnesota), and a few months after two white male vigilantes murdered Ahmaud Arbery, a Black man jogging outdoors (Georgia) and the police killing of Breonna Taylor, a Black woman sleeping, during a botched home invasion (Kentucky). These and other incidents of seemingly differential treatment of minoritized peoples (e.g., a tasing of a Native American man walking off-trail in a National Monument located in New Mexico; Salcedo, 2020), (re)elevated the recognition and attention on racial inequality in the U.S., including within nature-based leisure spaces.

Beyond fear of physical harm, constraints to participation in nature-based leisure for minoritized audiences include psychological harm due to discrimination and feeling unwelcomed by members of a dominant culture, as well as discomfort or disapproval by close confidantes (Gobster, 2002; Flood and McAvoy, 2007). For example, Shores et al. (2007) found that older Black women experienced disapproval from family or friends for participation in nature-based recreation concomitant with other constraints. Thus, members of minorized groups who engage in nature-based leisure may feel isolated (from those within their social network) alongside being minoritized (by those in the dominant group) for engaging in “white” activities despite ancestral ties to lands and nature for time immemorial (hooks, 2009; Savoy, 2015). Nature-based spaces, such as federal public lands in the U.S., tend to be maintained for leisure activities “preferred” by the dominant (white) culture which promotes nature as pristine, quiet, and in need of protection from man (Taylor, 2000; Finney, 2014). This narrative is not only exclusive in terms of leisure but erases the rich cultural traditions of Native peoples and their complex relationships with other entities (e.g., wildlife, plants, rivers) that occurred on lands occupied prior to decades of exclusion, extermination, false promises, and broken treaties by the U.S. government (Savoy, 2015). As evidenced by these examples, present-day concerns about psychological harms are multifaceted and can be entwined with deep-rooted trauma.

In the face of trauma, “transgressions” offer stories of hope and pride for minoritized communities in the context of nature (hooks, 2009; Theriault and Mowatt, 2020). Minoritized peoples have found safety, solace, community, freedom, food, and more in nature-based spaces–whether for purposes of leisure, work, sustenance, or other purposes. For example, thousands of Black people found opportunities in the Great Dismal Swamp (North Carolina) for employment (along the canal and in the forests) and to fight for (as part of the Union army during the U.S. Civil War) or escape to (as part of the Underground Railroad) their freedom during the 19th century (Maris-Wolf, 2002). Many people of color also intimately knew or came to understand the land and its non-human inhabitants for centuries through farming, harvesting and collecting plants or wildlife (terrestrial or marine) for sustenance (hooks, 2009), fire management practices (e.g., Roos et al., 2021), and more. Examples of “two-eyed seeing” (blending western scientific methodologies with traditional ecological knowledge) also demonstrates how Indigenous understanding of wildlife and land use can greatly improve understanding of complex systems (Housty et al., 2014; Rapp Learn, 2020; Henson et al., 2021). People of color have also engaged in leisure activities, such as camping, swimming, and traveling for pleasure, despite oppressive circumstances (Young Armstead, 2005; Wiltse, 2010; Kahrl, 2012; Algeo, 2013). These stories underscore the resistance and agency of minoritized populations who have remained committed to developing, maintaining, and sustaining connections to nature despite efforts by the dominant culture to exclude (or include) them, therefore offering opportunities for moving forward if these transgressions are fully heard and embraced.

Theriault and Mowatt (2020) challenged the narrative of nature as White by demonstrating how African Americans engaged with nature (which they term “wilderness”) in 4 specific ways that transgressed oppression during 3 specific time periods - (a) Enslavement, (b) Reconstruction and Post-Reconstruction, and (c) 1936–1994. The 4 transgressions offered by the authors included:

1. Wilderness as a space free of White oppression,
2. Wilderness as a site to challenge White oppression,
3. Engagement with wilderness despite White oppression, and
4. Advocacy for more just relationships with wilderness.

We extend these forms of transgression to how minoritized people more broadly may experience nature-based leisure, where nature is a space free of mostly social oppression (that causes or *re*-creates trauma), a site in which oppression or trauma (enforced by institutions) can be challenged, nature *re*-connection despite trauma, and advocacy for more just relationships with nature and other beings (e.g., wildlife, other people). Though we primarily focus on examples pertaining to racially- and ethnically-diverse peoples in this paper, our extension of Theriault and Mowatt's (2020) approach could apply to other minoritized groups (e.g., people minoritized based on physical or mental abilities, sex/gender, age, etc.).

### Co-cultural Theory to Understand the Experiences and Interests of Minoritized Groups on Public Lands

Given this background, co-cultural theory, which builds upon muted group theory (Kramarae, 1981) and standpoint theory (Smith, 1987), offers a useful perspective for framing analyses of traumas and transgressions related to nature-based leisure activities on public lands. Specifically, co-cultural theory supports an analytic strategy that seeks insight on the relations of dominant and non-dominant (i.e., co-cultural) groups from the perspective of minoritized peoples (Allison and Hibbler, 2004). Thus, co-cultural theory focuses attention on the experiences of people or groups *outside* the dominant culture to increase understanding of their needs and desires. Co-cultural theory also brings attention to possible ways that policies and procedures enacted by those with power in a particular domain, such as nature-based leisure spaces (Allison and Hibbler, 2004), do not address culture and social obstacles (e.g., trauma) experienced by those with less power in that same domain. Power can obfuscate understanding of the experiences of others (Orbe and Roberts, 2012) and obscure an “embedded nature of bias, stereotypes, and institutional discrimination that may exist” despite the best of intentions of powerholders (Allison and Hibbler, 2004, p. 262). For example, the policies of federal public lands determine what is acceptable nature-based leisure activities for visitors, and staff from the dominant culture reproduce stories encoded in those policies regarding “appropriate use” of natural resources as commodities (Martin, 2004; Finney, 2014; Floyd et al., 2014).

To date, several initiatives by federal public lands agencies seek to connect diverse urban audiences to nature-based leisure activities (e.g., the U.S. Fish and Wildlife Service's Urban Wildlife Conservation Program, the National Park Service's Urban Agenda, and the U.S. Forest Service's Urban Connections program). These programs aim to understand and overcome factors that inhibit connection of diverse urban audiences to biological diversity and nature-based leisure but may not expressly address historical traumas or uplift stories of transgressions that center the experiences of minoritized peoples, and these efforts may overlook the connections of diverse rural peoples as well. Floyd et al. (2014) noted that public lands agencies should “reflect on whether [their] programs and policies serve to increase access and promote freedom of choice or continue to maintain and reproduce inequalities and stereotypes” (p. 298). Leisure Constraints Theory suggests that institutional factors impacting preference and participation are typically embedded in the systems of the dominant culture, then trickle down from the institutional level to the interpersonal and intrapersonal levels (Jackson et al., 1993). Thus, the dominant culture influences co-cultural groups in the form of structural, interpersonal, and intrapersonal constraints (Crawford and Godbey, 1987), suggesting that systemic constraints embedded within the institution can reproduce as outward displays of other constraints. For example, someone saying they have “no interest in nature-based leisure” may actually be no patience for narratives that minimize the experiences of Black, Indigenous, and other peoples of color. Therefore, effective solutions that prioritize nature-based leisure as a human right require digging into and addressing the systemic roots of trauma, and uplifting stories of transgressions that center the experiences of co-cultural groups.

Our work extends beyond the constraints discourse by exploring the challenges faced by minoritized peoples who access nature-based leisure spaces. We specifically extend previous research exploring the themes of trauma and transgression in the context of nature-based leisure that focuses on the experiences of African Americans (e.g., Theriault and Mowatt, 2020) by examining the experiences of a broader range of people minoritized in the U.S. by race or ethnicity, and nationality in the case of Native Americans. This approach allows researchers and practitioners from countries that do not share the U.S.'s specific history of slavery and segregation to further apply Theriault and Mowatt's (2020) foundational ideas. Our use of co-cultural theory provides new insights into trauma and transgression by directly elevating the voices of people from minoritized backgrounds, identities, and/or communities and exploring how the dominant culture shapes the experiences of co-cultural groups in ways that cause (or reinforce) trauma, prevent healing, or require transgression. Thus, the methodological approach we undertook can also be used in places worldwide specifically because it uplifts stories of hope and perseverance in the face of oppression whether due to race and ethnicity, gender or sex, differing mental or physical abilities, body size differences, or more.

## Methods

Using this framework, we explore how historical traumas and transgressions surface as constraints to nature-based leisure. This work was part of an overall study conducted in collaboration with the U.S. Fish and Wildlife Service (FWS) to understand the beliefs about and experiences with nature-based leisure of diverse peoples living in urban communities proximate to National Wildlife Refuges (i.e., refuges). The study employed qualitative data collection and analysis using coded focus group methodology (Morgan and Krueger, 1993; Morgan, 1996; Krueger and Casey, 2009), with focus groups referred to as “community workshops” throughout the project.

### Site Selection

Potential locations for the community workshops were first identified from a list of refuges having 250,000 or more people living within a 25-mile radius of the refuge, the average distance a visitor traveled according to the National Visitor Survey conducted on refuges prior to this work (Sexton et al., 2012). Seventy-one refuges met the minimum criteria for inclusion, and the FWS prioritized a subset of those refuges to inform objectives of the Urban Wildlife Conservation Program (https://www.fws.gov/urban/index.php). Urban-proximate refuges were, in part, prioritized because the U.S. Census Bureau (2018) reports that 80.7% of U.S. residents live in urban areas, and these locations often have differential access and opportunities for nature-based leisure, which could be provided by urban refuges. Additionally, more people of color in the U.S. live in urban areas than do whites (Berube et al., 2010), and significantly more non-white people (74%) live in nature-deprived areas compared to white, non-Hispanic individuals (23%) living in similar areas (Landau et al., 2020). These statistics underscore a need to hear from minoritized people about their experiences with nature-based leisure in urban locations. Seven refuges were ultimately selected using purposive sampling that promoted maximum variability based on geographic location, cultural diversity, surrounding population size, visitation rates, and outreach efforts current to this work (Table 1). Maximum variation sampling is appropriate when the sample size (in this case, refuges) is very small (List, 2004) and when researchers want to understand how a phenomenon is seen and understood among different people, in different settings, and at different times (Cohen and Crabtree, 2006).

**Table 1:** National Wildlife Refuges (NWR) selected for participation in community workshops.

**Refuge**	**Urban Area(s) within 25 miles**	**Population within 25 miles**	Refuge Annual Visitation 2013^**^
Tualatin River NWR	Portland, OR-WA	1,727,100	131,709
Don Edwards San Francisco Bay NWR	San Francisco-Oakland, CA; San Jose, CA; Concord, CA	5,021,028	685,400
Rocky Mountain Arsenal NWR	Denver-Aurora, CO	2,277,371	180,000
Minnesota Valley NWR	Minneapolis-St. Paul, MN-WI	2,610,793	230,000
John Heinz NWR at Tinicum	Philadelphia, PA-NJ-DE-MD	3,949,328	140,000
Potomac River NWR Complex			
Featherstone NWR	Washington, DC-VA-MD	2,479,129	20
Mason Neck NWR	Washington, DC-VA-MD	2,832,706	38,210
Occoquan Bay NWR	Washington, DC-VA-MD	2,774,276	38,210
Arthur R Marshall Loxahatchee NWR	Miami, FL	2,586,378	276,680

* *Based on 2010 U.S. Census data (U.S. Census Bureau, 2010)*.

** *Based on 2013 Refuge Annual Performance Plan data (USFWS)*.

### Participant Selection

To recruit participants for the community workshops, the research team created an initial list of potential key contacts using online searches of organizations that served or represented minoritized groups^2^ in local communities adjacent to the selected refuges. Refuge staff also provided names and contacts for leaders or members of local organizations which serve minoritized populations, and community representatives familiar with local majority-minority neighborhoods. Next, we solicited input from initial contacts using a snowball sampling or chain referral technique to identify additional individuals and organizations to contact for participation. Finally, we conducted another internet search to find additional representatives from groups not already agreeing to participate. Similar to refuge selection, we used a maximum variation sampling approach based upon the type of organization that the individual represented or their role in a community. We used a set protocol and text template for initiating contacts and sending invitations and follow-up reminders to potential participants.

### Data Collection

At least one member of the research team facilitated each community workshop with assistance from two notetakers. Facilitators summarized and recorded comments in real-time on flip charts that could be viewed and further discussed by participants throughout the workshop to ensure accurate capture of key points. Participants had multiple opportunities to review and clarify statements, as well as to add missing information. One assistant recorded near verbatim notes on a laptop, identifying individual speakers with an anonymous coding system. Notes were reviewed and minimally edited for clarity immediately following the community workshop. To foster an open discussion and confidentiality, no audio or visual recording was used. The assistant taking notes remained the same at every community workshop, but the chart note-taker and facilitator varied depending on the location.

### Structure of Community Workshops

Two community workshops were held for ~2 hours each in a conference room or meeting space at the seven selected refuges. One workshop was held in the morning and the other was held in the evening of the same or next day (with the exception of the Rocky Mountain Arsenal, which was approximately 6 months apart), and participants only attended one of the two workshops at each site depending on which day/time best accommodated their availability. Participation was voluntary, and some refreshments (food or snacks and non-alcoholic drinks) were served. Otherwise, no money or other incentives were provided to participants.

To begin each workshop, participants were welcomed by the facilitator and refuge staff (if present); then all attendees introduced themselves and indicated the organization or community they represented or belonged to. Before the discussion of constraints^3^ began, any present staff members were excused from the room to encourage full disclosure of opinions about the refuge or FWS if warranted. Then, the facilitator reviewed the goal and guidelines for the workshop and began the discussion about nature-based leisure activities (i.e., outdoor recreation). Workshops were guided by the following questions, which were also advertised in advance of the meeting:

1. Speaking on behalf of local community residents, what comes to mind when they hear outdoor recreation?
2. What motivates people in this community to participate in outdoor recreation?
3. What barriers prevent greater access or enjoyment of outdoor recreation opportunities by people in this community?
4. What can be done to promote greater participation in outdoor recreation and use of the refuge by people in your community?

### Data Analysis

Data were analyzed using thematic analysis methods described by Benaquisto (2008) and Silverman and Patterson (2015). The goal of the thematic analysis was to explore the range of themes through use of explicit criteria and evidence from the data. Data analysis was systematic, verifiable, sequential, and continuous (Krueger and Casey, 2009). We used NVivo qualitative data analysis software to facilitate open and focused coding. Open coding (Krueger and Casey, 2009) involved reading the workshop notes, flip charts, and transcripts line-by-line and assigning codes to discrete excerpts from the text. Those codes could be drawn directly from the text or be researcher-generated based on the text. A single phrase could be assigned multiple codes. An initial coding frame or “start list” (Riddick and Russell, 2008) was developed from categories compiled by (Walker and Virden, 2005).

After initial coding, we then conducted focused coding (Krueger and Casey, 2009) in which the open codes were synthesized and organized into overarching categories or refined into more distinct variations. These focused codes were conceptual in nature and process oriented. Open and focused coding were repeated in an iterative fashion. Coding schemes were adjusted and the coding frame refined to inform the research questions, while also staying alert to other concepts that emerged (Krueger and Casey, 2009). The product of thematic analysis includes a description of the patterns of experience and the overarching design that unites those patterns (Ayres, 2008). The data analyst kept notes on insights, ideas, and patterns that occurred during the content analysis which were cross-referenced with the research team.

## Results

### Participants

Workshop participants included residents and representatives of more than 80 different communities or organizations, including organizations related to municipal parks and recreation in communities of color, community or urban development in underserved areas, faiths and religions important to co-cultural groups, education and small-scale childcare in communities of color, food justice for Indigenous communities, communities where environmental injustices had occurred, local radio and television geared towards speakers of languages other than English, youth groups (e.g., Boy Scouts, Girl Scouts, Boys and Girls Clubs), and more. Eleven of the 14 workshops had the ideal size of five to eight participants (Krueger and Casey, 2009); exceptions included a single workshop at Don Edwards San Francisco Bay (2 participants), Potomac River (3 participants), and Arthur R. Marshall Loxahatchee (16 participants).

### Constraints to Outdoor Recreation

Conversations around lack of resources, lack of awareness of the refuge, non-congruent outreach methods, and other well-documented constraints and common solutions uncovered examples of trauma and transgression in nature-based leisure. Results of the overall project are available elsewhere (Floyd et al., 2016); here, we present a summary of how trauma and transgression expressed by participants from racially- and ethnically-diverse peoples arose during discussions had at the community workshops.

### Trauma

*Trauma related to unequal treatment of people of color by authorities*. Participants voiced several concerns about employees of federal public lands wearing a uniform that may not be distinguishable from security forces (e.g., police, immigration and customs enforcement) that have stigmatized, minoritized, and at times brutalized people of color. Uniforms, coupled with fences, gates, signage, and other outreach material regarding regulations and statements of what to do or not do on refuges, promote an intimidating, authoritarian, or unwelcoming image about who belongs and who will be protected – or not.

*The [community is] used to seeing authority figures in their neighborhoods; when they see a ranger outdoors, they associate the staff with neighborhood authority figures and it is negative*. (Tualatin River)

*Some of these kids have seen parents arrested by someone in uniform, and they don't know the difference between a park ranger and an immigration officer*. (Don Edwards San Francisco Bay)

*Uniforms can be a barrier - actually scare some people*. (Rocky Mountain Arsenal)

*The big chain link fence… all you have to do is put razor wire over it and it looks like you're trying to keep us out or something in*. (Rocky Mountain Arsenal)

*There are many things that wildlife refuges are not for… trying to define all that you can and can't do is kind of difficult*. (Minnesota Valley)

*The signs tell you all the things you're not supposed to do. It's not exactly inviting*. (Potomac River)

*People from my community don't know [what's here on the refuge]. Think it's the Army and that they can't go on it. We're right there and don't use it… also don't want to be the only Black people out there… out in the woods by yourself*. (Rocky Mountain Arsenal)

*[The staff] put a great video together, very well done, but I asked them to watch and see how many persons of color are represented. We have a large population of Hispanics and Blacks [living in the local area] - not one person [in the video was a person of color]. Video says you're not welcome*. (Rocky Mountain Arsenal)

*Trauma related to occupation and class*. Participants from several workshops discussed the idea that families or groups of people historically lived or worked outside in tough circumstances (sometimes against their will), and many families want to demonstrate economic mobility and desire more for their children than they experienced during their own lifetimes. These beliefs included not wanting kids to work jobs or internships (particularly in cultures where the father is the sole financial provider for the family). Thus, spending time in outdoor settings may be perceived by some members of different communities as a socioeconomic backslide.

*Gardening, working, or spending time in the outdoors is a part of a heritage that people are trying to get away from*. (Tualatin River)

*I told my grandmother I was going to be a wildlife biologist and work outdoors. She said, “No, no, we worked too hard for you to be outdoors.” People equate working in an office with upper-level positions. There is still thinking like that among Latinos. It's a very real barrier… My husband's dad didn't think he had a real career because he was outside teaching kids to fish*. (Rocky Mountain Arsenal)

*In Peru, kids would never have summer jobs. That would be demeaning for the family. I was shocked and this was a real eye opener. In America, it's not demeaning, it's just good fun*. (Tualatin River)

*Trauma related to environmental racism*. Some participants noted trauma associated with how governing authorities and businesses treated the land and communities of color. For example, participants at Don Edwards San Francisco Bay noted that the smells and perceptions of toxicity from a garbage dump adjacent to the refuge promoted a negative view of nature-based leisure on the refuge. As another example, the majority Black community living south of the Rocky Mountain Arsenal historically experienced the area (prior to the refuge being established) as a chemical weapons manufacturing facility. The heavily polluted area made the National Priorities List as a hazardous waste site by the U.S. Environmental Protection Agency (EPA). During testing and investigation of the area, then-endangered bald eagles were found, prompting the establishment of the area as a refuge. Eventually, a fence was built around the refuge and the known entrance providing the historic community of color with access to the refuge was closed and a new visitor center and entrance were promoted, seemingly prioritizing a wealthy white community (see Sexton et al., 2015 for more). This community was named (until 2020) after the longest-serving mayor of Denver (Colorado) who also happened to be a member of the Ku Klux Klan (a white supremist group) for several years at the start of his career ostensibly for political gain (Goldberg, 1981).

*[Some reasons that] no one comes out here include… traffic, [lack of] transportation, location, the smell. People talk about the smell: “That's where the dump is.”* (Don Edwards San Francisco Bay)

*When we were growing up, we heard elected officials say, “Don't go to the refuge, the deer glow.” It was chemical pollution, not radioactive or nuclear. It's important from an environmental justice standpoint; historically, the refuge was not a safe place to be. Even up until recently, they are still monitoring pollution levels in nearby communities*. (Rocky Mountain Arsenal)

*The site served a purpose, to protect this country, but poisoned communities around here. People are not going to forget that*. (Rocky Mountain Arsenal)

*My children were young then. We would get calls for sarin warnings. It could hit while I was out in the yard with the children. The environmental justice component was not that long ago*. (Rocky Mountain Arsenal)

*Every time I bring up the refuge to people in my community, they bring up chemicals. [My community is] still mad. They think and say that the only reason [FWS] cleaned up [the site] is because they found some bald eagles*. (Rocky Mountain Arsenal)

*Trauma related to the Black experience*. Participants noted that nature-based areas, such as forests, can reinforce historical trauma and cultural stigmas (e.g., around crime and safety) for Black Americans in particular. These are places where past oppressive acts, including violence (e.g., lynching of Black Americans), were perpetrated, discrimination is still rampant, and many were warned by elders not to visit these places. Additionally, Black participants discussed judgement from white people for how they dressed, talked, etc.

*The only Black person in the gate doesn't want to be the only one out there. It's what you always remember – Black people are always the first killed*. (Rocky Mountain Arsenal)

*Fear also belongs to the parents because lynching happened in the woods*. (Minnesota Valley)

*Growing up I was not encouraged to go outdoors. Look at the historical reference point of people of color in the outdoors and what access and safety they have*. (Rocky Mountain Arsenal)

*My parents were telling me don't go into woods. You might see a friend hanging from a tree. Trying to escape slavery. Trying to get out of woods, away from those threats. Going back to the woods is a step back. There is a certain social history. People don't like to have that discussion. It just hits that nail on the head. For urban people, people of color, it's a challenge and I don't know how you overcome that*. (John Heinz)

*Next time you are in the mountains in a gas station see if you see any Black kids. When I pull up with a van of Black kids I have to say to clerk, I got this. They think they're going to steal, etc. If you're a young Black man in your 20s and you go up there, what comments do you think you would hear? It's not safe. The outdoors isn't what's unsafe, it's not safe to be around other people*. (Rocky Mountain Arsenal)

*Received an email from [another colleague] saying he was sick and tired of being judged. Getting judged on how he dressed, how he's talking, everything. All he wants to do is [a specific nature-based leisure activity]. People he's connecting with are not peers, but leaders. And he's being judged by them*. (Tualatin River)

*Trauma related to the experience of Indigenous peoples (i.e., Native Americans)*. In the U.S., tribal lands were stolen by the federal government through policies and practices or by others (e.g., colonizers, other residents) through brute force, leaving a legacy of distrust and reinforcing ideas of competition for the same resources. Additionally, Native perspectives on land rights and naming practices are often erased or ignored by the dominant culture, or further divides Native peoples because multiple tribes can have ties to the same land.

*The Native community nonprofits often compete with government and land management agencies [for funding]*. (Tualatin River)

*[Native communities have] historical trauma, being pulled from the land, whether from Oregon or other places in U.S. during relocation. Historical policies are in place to disconnect Native Americans from homeland and assimilate*. (Tualatin River)

*[We have a] cultural perspective of families first [in our community]. No one else is trusted*. (Tualatin River)

*[We have an] interest in [nature-based leisure] areas reflecting what was historically here. To honor Native plants and an ability to forage for native foods. [We would like] recognition and naming of these areas with traditional names. Native American community appreciates it. [However, we can] get into political arguments about what to name it because the land is associated with different tribes*. (Tualatin River).

### Transgression in the Face of Trauma and Oppression

Participants also voiced ways in which they or people they knew had transgressed traumas and constraints on nature-based leisure activities. In fact, for several participants, their participation in these workshops was an act of transgression, with some individuals noting that their presence has not always been invited or welcomed by authorities at previous publicly open meetings, or were not honored when they did speak up; however, these participants expressed continued dedication to making change and were passionate about the subject matter regardless of past experiences.

We present below some example quotes to demonstrate and expand upon the 4 types of transgression outlined by Theriault and Mowatt (2020), who wrote about the experience of American Americans and nature-based leisure. Our results showcase how their foundational framework can be applied to groups of people who have been marginalized by the dominant culture in any nation or by any institution that enforces who belongs and what is “appropriate” in public spaces where nature-based leisure occurs.

1 *[Nature] as a space free of [social] oppression*. Theriault and Mowatt (2020) describe how African Americans created safe spaces in wilderness outside of white oppression across three historic time periods. We extend the meaning of this transgression to recognize that engagement with nature by minoritized peoples can provide freedom from oppression (i.e., being told what you can and can't do) by those in the dominant culture. As an illustration, community workshop participants spoke about how engaging in nature-based leisure or being on the refuge allowed them to escape social control and troubles (e.g., stress, worries, crime, sirens) associated with urban life.

*Very clear that kids prefer this environment [the refuge] to any playground in town. We do go to all the playgrounds–and the kids say “Can we go back to the woods? Can we go back to creek?” Their reaction when we get [to the refuge] is ‘YAY!’ All run off to their level of participation*. (Don Edwards San Francisco Bay)

*The outdoors is a healing ground… a sacred place [of] nature and self-discovery*. (Tualatin River)

*The benefits [of nature] are a sense of peace and being away from the hustle and bustle, crime, and sirens*. (Minnesota Valley)

*Children don't want…to be told everything. They can merge with nature. They just need space to play and they will hide, climb, crawl, and explore. For preschool maybe up to age 7, just show them an environment full of nature—they just want to play and want freedom*. (Loxahatchee)

*The outdoors has a spiritual quality. You can wash away stress and worries*. (Potomac River)

*Refugees [immigrants] from all over, they didn't come by choice, but as a by-product of foreign policy. Nature is healing*. (Tualatin River)

2 *[Nature] as a site to challenge [institutional] oppression*. Theriault and Mowatt (2020) describe historical examples of ways in which African Americans directly challenged oppression as well. Here, we expand on this idea to showcase how minoritized peoples directly challenge the efforts of institutions and powerholders (e.g., public lands agencies, policing authorities, policy regulators) to shape their behavior. Thus, this transgression signifies a desire to overcome dominant expectations about who does and doesn't belong or what can and can't be done in public spaces. Control efforts included those that are formal (e.g., signage, fencing, policies, charging fees for entrance) and informal (e.g., frowning, verbal confrontation).

*The fence [when my husband was young] was horrible and they would climb over and play [in nature despite sarin gas warnings] because there wasn't any open space in the community*. (Rocky Mountain Arsenal)

*Go out to Columbia Gorge and drive along old highway. Look at ethnic diversity of all people stopping at the sites. Then go to the biggest and most accessible [and free] one… 1*st *or 2*nd *most visited place in the state. Quite remarkable, like the United Nations, so many languages being spoken – delightful*. (Tualatin River)

*Even out at campsites, when our culture comes, people don't understand us or they think, “We have to watch them, they drink.” A lot of people drink – it's not just our culture. Or, they think, “Oh, they will play music loud.” A lot of people play music loud. It's important for rangers, whomever, to be intentional about getting different cultures out to these places to make the places feel inclusive to everyone*. (Don Edwards San Francisco Bay)

*What [parents] see on the 6 o'clock news and on the corner puts them in a different mindset. Always looking for cops; if someone gets hurt, [the situation] turns into a fiasco–[when I was growing up], issues were settled between parents and kids. Now you go to [a juvenile detention center] for 4–5 years [for the same behavior we did as kids]. The opportunity [to learn from mistakes] is no longer here. [Nature can help] create unstructured moments within safe environments [to shoot BB guns, throw rocks, and otherwise learn right and wrong]*. (Don Edwards San Francisco Bay)

3 *Engagement with [nature] despite oppression*. Theriault and Mowatt (2020) describe how African Americans have continued to engage with nature despite very real threats to their safety. With respect to this idea, we offer quotes from participants who spoke of having transgressed oppression by engaging with nature despite historical trauma or ongoing (externalized and internalized) discrimination that affects beliefs about who can be in nature. In some cases, being in nature was described as best done alongside people who fit the same demographic(s) and mindset, essentially suggesting a “strength in numbers” perspective to counter feelings of discomfort associated with spaces “normalized” as white (Kobayashi and Peake, 2000).

*When I do outdoor activities the kids in our programs are like “You do archery? You hunt?” And when there are other Black instructors, they're like “You do this?” They don't expect Black people to be doing these things*. (Don Edwards San Francisco Bay)

*Especially African Americans have a fear of water–[there's a] stereotype that African Americans can't swim. Really not true… [Our group is] talking with the coast guard about teaching basic water skills to pierce that fear. Fear is what kills you in any situation. Need to understand that water is a friend. Certain technique to stand in a flowing current of water [when fly fishing]. Lean back like in a chair, if you stand up, the river pushes you over; need to use the current to your advantage. Bringing in coast guard to teach basic water survival techniques also gives coast guard an opportunity to connect with the group [of kids from minoritized backgrounds]*. (Tualatin River)

*For Native Americans, [there is] cultural motivation. [Nature is] a place to put on events, pow-wows, gatherings*. (Tualatin River)

*[If you've ever been racially profiled, you] feel like people are watching or looking at you [when you spend time in nature surrounded by white people]. You feel like an outsider and alienated. If I come to the refuge and see people riding $800 bikes with all the gear, equipment, clothing, etc., I feel like an outsider. So, being able to have someone else [that looks like me] shepherd you in and get you acclimated [to nature], makes you realize not everyone is looking at you*. (Don Edwards San Francisco Bay)

4 *Advocacy for more just relationships with [nature]*. Finally, Theriault and Mowatt (2020) underscore that African Americans have continuously advocated for environmental justice. We expand this idea to include the ways in which minoritized populations have and continue to advocate for just relationships with the land and other beings, such as wildlife and humans from different cultural backgrounds (taking the viewpoint that humans are part of nature). This advocacy includes understanding and articulating past traumas to nature through pollution (so as not to forget and to encourage restitution) as well as how different peoples have worked with the land (e.g., through farming practices, burning with fire, etc.) and used natural resources (e.g., native plants, wildlife) for time immemorial. Additionally, several participants at one community workshop discussed how engagement with nature allowed for improved understanding of more just relationships with all life forms.

*From historical standpoint, a lot of people would like to do away with [the refuge name] because of what happened here. But from an environmental justice standpoint, until there is appropriate recognition of what happened, it's important*. (Rocky Mountain Arsenal)

*If we are creating ways to get students involved using outdoor connection through careers, we have to make activities, especially service-learning activities, multigenerational. There should be an educational component, not just showing birds and places, but using traditional ecological knowledge (TEK) where you're able to make a holistic connection*. (Tualatin River)

*It's a wildlife refuge, so the first concern is to protect the refuge. But, you have man-made ponds with non-native [fish] species. Really think about that. Nonnative flora and fauna. You're protecting a non-native resource. [Also, some of the staff] here have tunnel vision: protect, protect, protect. Not thinking about teaching the public to protect. [Instead, the staff think] they're responsible for protecting it*. (Rocky Mountain Arsenal)

*[Some youth who had difficult childhoods] saw catfish be cleaned and eaten. It gave them an opportunity to address hard hitting questions that they think, but don't feel comfortable asking. We're pulling skin off catfish and when finished I state, “that's what you see in the grocery store.” All through that process they ask questions – hard hitting questions, like “What's the difference between that and murder?” I explain that it's lawful versus unlawful, and that this fish is going to be eaten for nourishment. Then they don't spout off and say, “I'm going to kill you” when playing, they think about it more, the cycle of life, and they make those connections they otherwise don't see every day. Really what we're trying to go for [by being in nature] is what it means to live responsibly in a social environment*. (Don Edwards San Francisco Bay)

## Discussion

This study invited residents and organizational representatives from minoritized backgrounds, identities, or communities to voice constraints regarding nature-based leisure activities (e.g., hiking, wildlife observation, hunting, fishing, birding) as part of workshops held on seven urban-proximate wildlife refuges in the U.S. Themes uncovered from the qualitative methodology underscored tenets of co-cultural theory, which suggests that the dominant culture shapes the experiences of co-cultural (i.e., minoritized) groups in ways that cause (or reinforce) trauma, prevent healing, or require transgression. In particular, numerous participants noted how the conditions that had caused trauma were overtly ignored or reinforced by institutions, creating subsequent effects on how people interact with each other (e.g., by profiling and stereotyping) and how one sees themselves in these spaces (e.g., feeling unwelcomed and unable or uninterested due to fear/discomfort). Themes of marginality, not feeling welcome or a sense of belonging, costs related to equipment and access, and cultural relevancy of staffing and public lands overall are well documented concerns among recreationists from minoritized backgrounds on a variety of public lands (Floyd et al., 1994; Taylor, 2000; Floyd, 2001; Lee et al., 2001; Finney, 2014; Krymkowski et al., 2014; Scott and Lee, 2018). However, researchers and practitioners might find more opportunities for change in listening to and understanding the ways in which minoritized peoples transgress socialized oppression and overt constraints (Theriault and Mowatt, 2020). In combination, these results suggest a significant need for action that addresses the traumas related to the institutions that perpetuate them (knowingly or not) and uplifts stories of transgressions that highlight the power of nature-based connections for these peoples.

These findings document the ongoing legacy effects of trauma from past practices and institutionalized discrimination, as well as beliefs about who and what is allowed and how people should act or be treated. Thus, historical trauma still affects the participation and preferences for nature-based leisure among minoritized peoples. Public lands with extensive, and often traumatic, histories can empower the voices of the communities they seek to reach by uplifting stories of personal (human) agency and community persistence (i.e., “transgressions”; Theriault and Mowatt, 2020) in addition to showcasing the natural splendor of the site (Sexton et al., 2015). Minoritized peoples also have insight that could inform decisions about public lands beyond leisure use (e.g., Housty et al., 2014; Lund, 2014; Rapp Learn, 2020) or that can minimize potential harm and lead to improved outcomes for their communities. For example, a permitting system (e.g., to reserve a campsite) to access many of America's public lands benefits the institutions that administer the system at the expense of people who distrust how those systems could be used to profile and minoritize (i.e., stigmatize) communities (Johnson et al., 2007; Walker, 2019). Understanding these constraints prior to making decisions can potentially circumvent them from happening or generate new outcomes that benefit those who might otherwise be minoritized by their implementation (e.g., funds saved by this new permitting system could be used toward free access passes for low-income recreationists or as a form of reparations that acknowledge stolen lands).

Participants also expressed an approach consistent with the United Nations (2016), essentially encouraging efforts to meet families where they are already engaged in leisure activities in trusted places they know, whether outdoors or, for example, in faith-based institutions (Walker, 2019). For many people of color (e.g., Latino, Middle Eastern, and Native American families), programming with a social and multi-generational component that centers the experience of the relevant cultures first can help build positive relationships with leisure staff and develop trust around participation in institutionally-run activities. Additionally, institutions that engage in active recruitment, creating a welcoming and trauma-informed environment, and culturally-informed advertising can help create a sense of welcoming and belonging. Dialogues with local communities about their needs and interests can further expand upon these results and their meaning within those locations. As a participant in one of the Tualatin River workshops mentioned, “There is incredible diversity within every community mentioned here today. We need to be careful not to assume that a connection with one person and what they tell us is generalizable to the whole community.” Therefore, methodologies based in co-cultural theory are critical for allowing researchers and practitioners to learn from people continually sidelined by power dynamics and truly listen to what matters to marginalized community members.

### Limitations

This study has several limitations. First, individuals recruited for some workshops did not always reflect the full diversity of demographics within the local population. Some invitations to participate went unanswered or were outright turned down (e.g., “the distance is too far for us to bother”). Similarly, a second limitation is that these groups were heterogeneous rather than homogenous, which likely affected the discussion of constraints. Thus, the degree to which traumas and transgressions were raised and discussed could differ depending on the composition of participants in a workshop. Third, some community workshops relied upon referrals by refuge staff more than others, which suggests an established relationship and raises questions about which group a person was part of (the “dominant” or a co-cultural group, as described by co-cultural theory). Additionally, the workshops were conducted on the wildlife refuge, which likely affected who participated as well as the way in which people engaged (e.g., acquiescence to group opinion can be influenced by setting attributes). Fourth, the facilitator of the workshops varied, and their communication styles, presentation, and demographics could have influenced the flow and content of the discussion at each location regardless of the set protocol that guided the study. Finally, the researcher who conducted the preliminary data analysis was present for three of the seven workshops; thus, some content may have been lost or misinterpreted across researchers and time given that some content (e.g., nonverbal cues of agreement or disagreement) was not transcribed (Krueger and Casey, 2009).

### Conclusion

Here, we specifically explored constraints related to historical trauma and types of transgressions that are often overlooked or ignored by the dominant U.S. culture, despite being routinely expressed and experienced by minoritized groups (in this case, racially- and ethnically-diverse groups). In the U.S., the dominant culture typically reproduces ideas of nature as quiet, pristine, and in need of protection–and primarily intended for white, if not also wealthy, audiences (Taylor, 2000; Finney, 2014; Scott and Lee, 2018). This thinking contradicts efforts to embrace “new” audiences and “diversify” the outdoors, which will require an expanded view of what nature-based leisure can be and who belongs in these spaces (Taylor, 2000). The recent growth and formalized interest of institutions, such as federal public lands management agencies, in adapting to changing social and environmental conditions in various ways suggests that the time for leisure to be realized as a human right among all groups of people is overdue and now. However, eradicating historical and current constraints will take serious effort on the part of institutions, which are often resistant to change and at times disconnected from local users' idealized nature-based amenities (e.g., de Bell et al., 2018).

Addressing historical traumas does not require centering the dominant culture (or institutions) as a “rescuer”; instead, embracing, financially supporting, and promoting the ways in which people from minoritized groups have traditionally and currently create and expand access may produce the permanent and powerful changes that ultimately ensure that public lands (and the health benefits they provide through leisure) are available for everyone to enjoy (Chavez, 2000). For example, organizations that currently prioritize the dominant culture can instead uplift stories of historical and current transgressions of minoritized peoples, and support the efforts of affinity groups to get people outdoors, create community, and educate others. Contemporary examples could include highlighting the leaders and participants of organizations such as Outdoor Afro (https://outdoorafro.com/), Latinos Outdoors (https://latinooutdoors.org/), Native Women's Wilderness (https://www.nativewomenswilderness.org/) and Indigenous Women Outdoors (https://www.indigenouswomenoutdoors.ca/), and People of the Global Majority in the Outdoors, Nature, and the Environment (https://www.pgmone.org/). Additionally, reading groups, such as The Joy Trip Reading project (https://joytripproject.com/the-joy-trip-reading-project/) uplift the voices and experiences of authors from traditionally minoritized backgrounds in literature as well as nature-based leisure spaces. Finally, efforts such as the National Wildlife Federation's Safe Spaces Initiative (https://www.nwf.org/Get-Involved/Events/Safe-Spaces) highlight numerous Black academics, natural resource managers, business leaders, outdoor adventures, and more, offering important learning opportunities for individuals, communities, and institutions on how to face historical traumas and share stories of transgressions in ways that center the voices and experiences of people of color. For example, recent survey data demonstrated that 42% of Black Americans and other people of color feel more welcome in campgrounds and outdoor settings than in the past (Cairn Consulting Group, 2017). These examples show powerful ways for creating and expanding access and represent a continuation of agency and transgressions that have occurred historically (Theriault and Mowatt, 2020), demonstrating how nature-based leisure, and the mental and physical health benefits these opportunities provide, can be a human right truly realized by all peoples.

## Data Availability Statement

Results presented here reflect the voices of participants from communities proximate to urban refuges and sharing of raw data may compromise the anonymity of participant voices. Thus, raw data are not readily available and requests to access data from this research should be directed to the corresponding author.

## Ethics Statement

The studies involving human participants were reviewed and approved by North Carolina State University, Office of Research and Innovation. Written informed consent for participation was not required for this study in accordance with the national legislation and the institutional requirements.

## Author Contributions

AD, MF, DR-W, and NS developed the research protocols with others. AD, MF, and DR-W conducted the research with the support of others. AD, MF, and EJ framed the paper. AD wrote bulk of manuscript with contributions from EJ and edits from co-authors. All authors contributed to the article and approved the submitted version.

## Funding

This research was funded by the U.S. Fish and Wildlife Service (Service) through Cooperative Agreement Number F13AC00174 Piedmont-South Atlantic Coast Cooperative Ecosystem Studies Unit (with North Carolina State University) and Reimbursable Agreement Number 4500049958 (with the U.S. Geological Survey).

## Author Disclaimer

The findings and conclusions in this article are those of the authors and do not necessarily represent the views of the U.S. Fish and Wildlife Service.

## Conflict of Interest

The authors declare that the research was conducted in the absence of any commercial or financial relationships that could be construed as a potential conflict of interest.

## Publisher's Note

All claims expressed in this article are solely those of the authors and do not necessarily represent those of their affiliated organizations, or those of the publisher, the editors and the reviewers. Any product that may be evaluated in this article, or claim that may be made by its manufacturer, is not guaranteed or endorsed by the publisher.

## Footnotes

^1^For an in-depth investigation of universities' profiteering from Native land dispossessions, see *Land-Grab Universities: A High Country News Investigation* (https://www.landgrabu.org/).

^2^Though we focus on racially- and ethnically-minoritized peoples in this paper, the broader research also sought input from people of other backgrounds minoritized in the U.S., including reaching out to organizations that serve populations with physical differences (e.g., vison or hearing difficulties, mobility challenges), worship non-dominant religions (e.g., Muslim, Jewish), or work with communities experiencing extreme poverty. Additionally, people have intersectional identities, meaning that any single person could have multiple minoritized identities.

^3^In the community workshops, we exclusively used the term “barrier” to avoid academic debates over the conceptual differences between barriers and constraints. However, throughout the text here, we exclusively use the term “constraints” since we are focused on what affects participation and preferences.
